# A compact system for simultaneous stimulation and recording for closed-loop myoelectric control

**DOI:** 10.1186/s12984-021-00877-5

**Published:** 2021-05-25

**Authors:** Martin A. Garenfeld, Nikola Jorgovanovic, Vojin Ilic, Matija Strbac, Milica Isakovic, Jakob L. Dideriksen, Strahinja Dosen

**Affiliations:** 1 Department of Health Science and Technology, Aalborg University, Frederik Bajers Vej 7D, 9220 Aalborg Ø, Denmark; 2 Tecnalia Serbia Ltd., Deligradska 9/39, 11000 Belgrade, Serbia; 3 Department of Computing and Control Engineering, Faculty of Technical Sciences, University of Novi Sad, Trg Dositeja Obradovica 6, 21000 Novi Sad, Serbia

**Keywords:** Closed-loop control, Myoelectric prosthesis, Electrotactile stimulation, Sensory feedback, Dynamic blanking

## Abstract

**Background:**

Despite important advancements in control and mechatronics of myoelectric prostheses, the communication between the user and his/her bionic limb is still unidirectional, as these systems do not provide somatosensory feedback. Electrotactile stimulation is an attractive technology to close the control loop since it allows flexible modulation of multiple parameters and compact interface design via multi-pad electrodes. However, the stimulation interferes with the recording of myoelectric signals and this can be detrimental to control.

**Methods:**

We present a novel compact solution for simultaneous recording and stimulation through dynamic blanking of stimulation artefacts. To test the system, a feedback coding scheme communicating wrist rotation and hand aperture was developed specifically to stress the myoelectric control while still providing meaningful information to the subjects. Ten subjects participated in an experiment, where the quality of closed-loop myoelectric control was assessed by controlling a cursor in a two degrees of freedom target-reaching task. The benchmark performance with visual feedback was compared to that achieved by combining visual feedback and electrotactile stimulation as well as by using electrotactile feedback only.

**Results:**

There was no significant difference in performance between visual and combined feedback condition with regards to successfully reached targets, time to reach a target, path efficiency and the number of overshoots. Therefore, the quality of myoelectric control was preserved in spite of the stimulation. As expected, the tactile condition was significantly poorer in completion rate (100/4% and 78/25% for combined and tactile condition, respectively) and time to reach a target (9/2 s and 13/4 s for combined and tactile condition, respectively). However, the performance in the tactile condition was still good, with no significant difference in path efficiency (38/8%) and the number of overshoots (0.5/0.4 overshoots), indicating that the stimulation was meaningful for the subjects and useful for closed-loop control.

**Conclusions:**

Overall, the results demonstrated that the developed system can provide robust closed-loop control using electrotactile stimulation. The system supports different encoding schemes and allows placing the recording and stimulation electrodes next to each other. This is an important step towards an integrated solution where the developed unit will be embedded into a prosthetic socket.

## Background

Even though myoelectric prostheses can partially restore lost functionality following transradial amputations or congenital limb deficiency, approximately 25% of the users abandon their prosthesis [1]. In addition to the requirements related to ergonomics and control [2], users also report the restoration of exteroceptive and proprioceptive feedback as an important future goal [3]. In the absence of somatosensory information, the users need to rely on visual and/or auditory input, which can provide a good estimate of the prosthesis state [4], but requires the subject to direct the attention to the prosthesis. A functional prosthesis without feedback can be viewed as an alien object; a mechanical tool required to accomplish the task rather than a bionic replacement of the missing limb. In fact, a sensory intact residual limb is in many cases more valuable to the user [5]. By closing the loop, it is expected that the users can reach a higher embodiment of the prosthesis since the need for visual attention will be reduced [6, 7]. Although demonstrating the functional benefits of feedback is not an easy task [8], several recent studies have indeed shown that providing sensory feedback can improve user performance [9–11] as well as experience [12, 13]. Currently, only three commercial prostheses promise to deliver non-invasive tactile feedback, namely, Vincent Evolution 4 (Vincent Systems, Karlsruhe, Germany) [14], Luke Arm (Mobius Bionics, Manchester, USA) [15] and Ability Hand (Psyonic, Champaign, USA) [16]; however, the benefits of feedback in these devices are yet to be clinically proven.

The feedback can be delivered either invasively or on the surface of the skin through various modalities [5, 17–19]. Invasive methods based on electrical stimulation of peripheral nerves [9, 20], spinal cord [21] and/or brain [22] can evoke somatotopic sensations, but they also demand a surgical procedure, and amputees can be reluctant to undergo additional invasive interventions [23]. A non-invasive approach is substitution feedback, which transmits the missing sensory information through another modality applied to a different site of the skin. Vibrotactile stimulation [24] utilizes small vibration motors to activate mechanoreceptors in the skin, whereas electrotactile stimulation [25] activates superficial nerves in the skin directly via surface electrodes. Although electrical stimulation can produce uncomfortable sensations if the parameters are not adjusted properly, it holds several advantages including low power consumption, independent parameter modulation and compact as well as flexible design [26, 27]. This can be particularly advantageous when using electrode arrays to present multiple feedback variables intuitively to the user [28].

An important drawback, however, when using electrotactile stimulation in closed-loop myoelectric control is that it interferes with the recorded myoelectric signal. The stimulation produces strong artifacts that can corrupt the signal and possibly saturate the electromyography (EMG) amplifier, leading to problems in prosthesis control.

The suppression of stimulation artifacts is a known and longstanding problem in the field of functional electrical stimulation (FES). In FES, the electrical stimulation is delivered to activate muscles and restore movements in paralyzed patients, and it can be combined with EMG to trigger the stimulation by detecting subject movement intention. The influence of spatial filters, electrode distance, and stimulation waveforms on the stimulation artifacts were evaluated in [29]. Software [30] and hardware blanking [31] as well as adaptive methods have been tested for artifact suppression [32, 33]. A specific challenge in FES is that the artifact also includes muscle electrical activity (M-wave) that is evoked by the stimulation itself and needs to be discriminated from the voluntary EMG.

Contrary to FES, the electrical stimulation in prosthetics is used to produce tactile sensations, which means that the stimulation is delivered below the motor threshold, hence no M-wave will be generated. Moreover, the pulse amplitude is lower and the current flow more localized, leading to artefacts of smaller amplitude. Nevertheless, the myoelectric control is also more sophisticated and relies on multichannel EMG and machine learning (classification and regression) to discriminate different movement patterns [34]. Several recent studies that employed electrotactile feedback avoided the interference by placing the recording and stimulation interface contralaterally [28, 35] or ipsilaterally but with large spatial separation (e.g., recording from the forearm while stimulating the upper arm [36]). This allows testing in laboratory but it is not applicable clinically, where both recording and stimulation have to be fitted within a confined area of the residual limb and prosthetic socket. This problem is exacerbated by the fact that both interfaces often include multiple channels (electrode arrays or matrices).

Only few studies have explicitly investigated the effect of electrotactile stimulation on myoelectric control. The presence of stimulation artefacts has shown to dramatically decrease the control performance when using an LDA classifier [37] and neural networks [38]. Delivering electrical stimulation more than 60 mm from the myoelectric recording site has shown not to interfere significantly with the control system [39]. Time-division multiplexing [40] and software blanking of stimulation artefacts [37] have been applied to allow simultaneous recording and stimulation without spatial separation between the electrodes. The former method divides stimulation time into on and off intervals (i.e., stimulation and recording windows), while continuously acquiring myoelectric signals. The contaminated signal (stimulation window) is then discarded and the artefact-free signal (recording window) is used for myoelectric control. While providing stable control, this approach does not allow a constant flow of feedback. Artefact blanking is a more selective approach, where the recording samples affected by an artefact after each electrical pulse are set to zero. For myoelectric control, the incoming EMG data are segmented for feature extraction [41]. In [37], various data segmentation strategies were applied to evaluate the classification accuracy of software blanked data sets. Even though the evaluation was performed offline, the same principles can be introduced in an online application by using a single device that integrates both a recording and stimulation unit.

The present study describes a novel system specifically designed for simultaneous electrotactile stimulation (16 channels) and myoelectric control (8 channels) with compact form factor (henceforth referred to as the MaxSens system). The system is described in detail, including hardware components and control algorithm with integrated dynamic blanking, and it was evaluated experimentally by assessing the quality of online myoelectric control with and without concomitant electrotactile stimulation. The electrotactile feedback was designed to include simultaneous modulation of multiple stimulation parameters to maximally “stress” the recording and classification system. The experimental evaluation confirmed successful application: online control was not compromised when activating stimulation and the tactile feedback was useful for the subjects when vision was unavailable. Therefore, the system recorded EMG of good quality while generating stimulation that the subjects could perceive and exploit for closed-loop control. These are encouraging results regarding the prospect of embedding the developed compact system in a myoelectric prosthesis.

## System description

### Electronic design

**Fig. 1:**
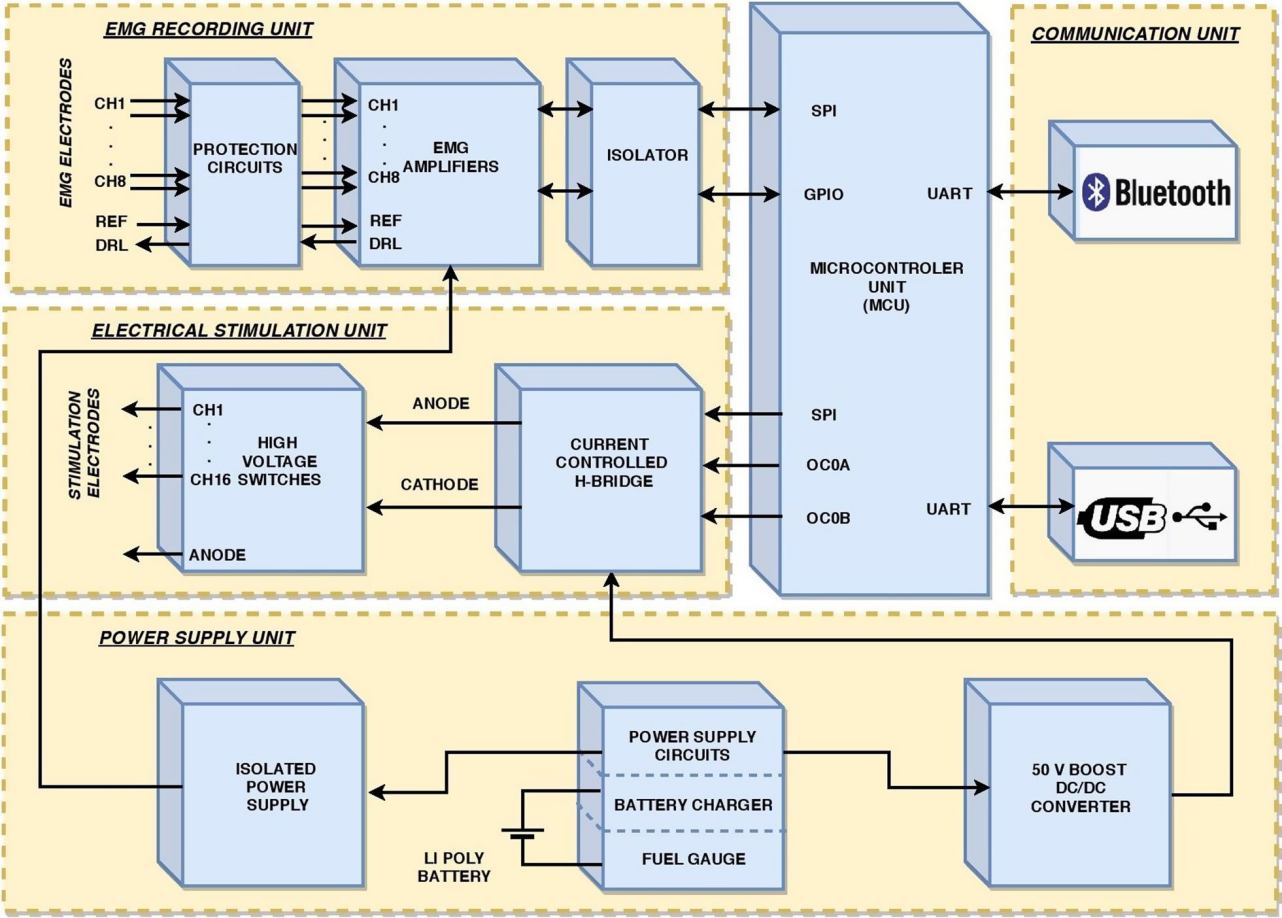
Block diagram of the units comprising the MaxSens stimulation and recording system (i.e., microcontroller, electrical stimulation, EMG recording, communication and power supply unit)

The MaxSens system is based on the concept of spatio-temporal distribution of stimulation pulses that was introduced for neurorehabilitation applications [42], and more recently proposed as an integrated and flexible solution for providing electrotactile feedback in the MaxSens stimulator prototype [27]. Further development of the system involved miniaturization of the device and adding features that resulted in a first standalone electrotactile closed-loop system that enables simultaneous recording and stimulation. The current system was briefly presented in a short conference publication [43]. The architecture of the system is shown in Fig. 1. As illustrated in this figure, the device consists of several independent units: microcontroller, electrical stimulation, EMG recording, communication and power supply.

#### Microcontroller unit

The microcontroller unit (MCU) is the central part of the MaxSens system. The MCU controls and synchronizes the other components of the device. It is based on an 8-bit RISC microcontroller ATxmega384C3 (Microchip Technology, Arizona, USA) integrating a rich set of hardware peripherals and memory resources. The MCU operating clock frequency is 32 MHz. The connection between the MCU and the electrical stimulation unit is implemented using a serial peripheral interface (SPI) communication module and two compare modules. The SPI interface is used for accessing the digital/analogue (DA) converter in order to set the amplitude of the current pulses and control the high-voltage switches to select active cathode fields on a 16-channel matrix electrode (see below). The two compare modules (OC0x) equipped with two 16-bit timers control the current source (H-bridge) to produce biphasic current pulses.

The connection between the MCU and the EMG recording unit is implemented using an SPI module and general-purpose input/output pins (GPIO). The SPI module is used for setting the desired parameters (sample frequency and/or EMG gain) of the EMG unit and for reading recorded EMG samples. The GPIOs control the timing of the sampling process. The connection between the MCU and the communication unit is implemented using two USART modules. Through these modules, the MCU communicates with the USB and Bluetooth controllers to simultaneously receive stimulation commands and transmit recorded EMG samples to the host computer.

#### Electrical stimulation

**Fig. 2:**
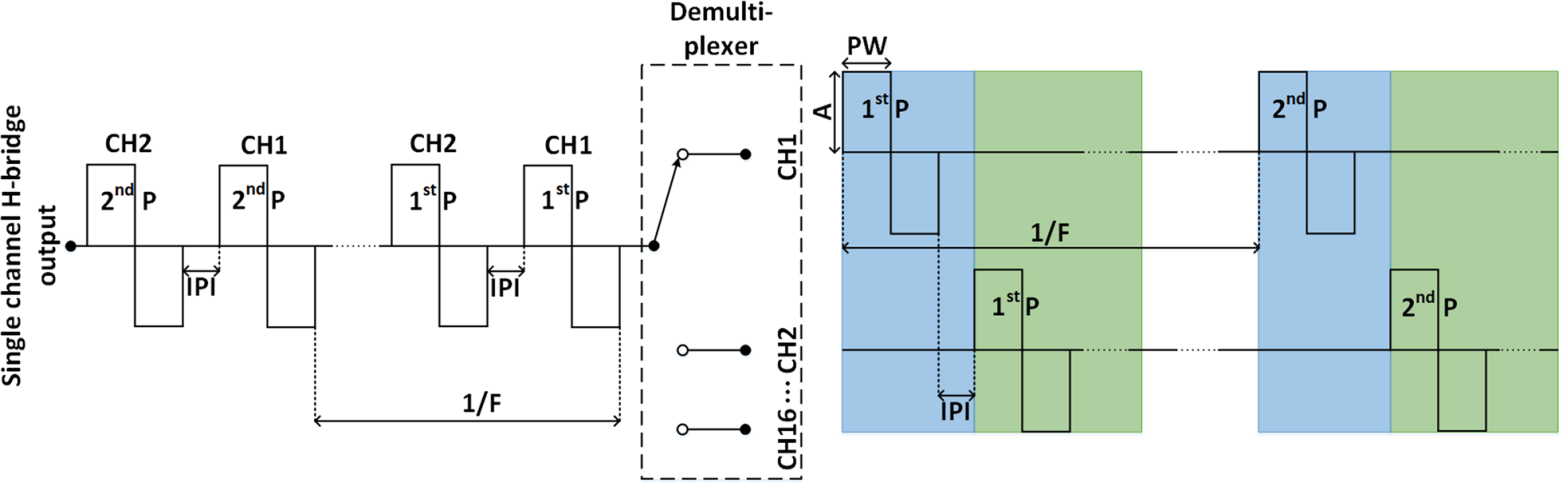
Illustration of the asynchronous stimulation protocol for a two-channel stimulation scenario, where IPI, F, P, PW, A and CH indicate inter-pulse interval, frequency, pulse, pulse width, amplitude and stimulation channel, respectively. The H-bridge outputs a multiplexed signal that is demultiplexed to the respective stimulation channels. All paremeters (IPI, F, PW and A) are equal before and after demultiplexing. The horizontal lines represents time, where the dotted horizontal segments indicate prolonged duration. The green and blue color indicate the blanking intervals, where the green color indicates blanking duration calculated from Eq. 1 or the first term within the brackets of Eq. 2 and the blue color indicates blanking duration calculated from the second term within the brackets of Eq. 2

The electrical stimulation unit is implemented as a current controlled H-bridge in order to produce biphasic current pulses. The H-bridge acts as a single channel stimulator generating a sequence of current pulses that are then distributed to matrix electrodes through 16-channel analog switches MAX14082 (Maxim Integrated, California, USA). The amplitudes of the current pulses are controlled by a 16-bit DA converter, while duration and frequency are set using the MCU compare modules and timers. The pulse width and amplitude can be adjusted for individual electrode pads in a range of 50–1000 µs with 10 µs steps and 50–10,000 µA with 0.1 µA steps, respectively. The stimulation frequency can be modulated in a 1–400 Hz range with 1 Hz steps and is common for all electrode pads. In each stimulation period, the pulses are generated sequentially and then distributed with adjustable inter-pulse interval (1–25 ms in steps of 1 ms) to all active stimulation channels, implementing a so-called asynchronous stimulation protocol. An illustration of the stimulation parameters and the asynchronous protocol in the case of two-channels is shown in Fig. 2. This approach is convenient for implementation, but it also enforces some constraints on the allowed combination of parameter values. Specifically, all pulses that should be distributed to active channels within a single period need to “fit” within that time interval. For instance, if we assume that all pulses have the same width, the following inequality must hold: ${\mathrm{N}}_{\mathrm{ch}}\cdot \left(2 \cdot {\mathrm{T}}_{\mathrm{pulse}}+ {\mathrm{T}}_{\mathrm{TBP}}\right)\le \frac{1000}{{\mathrm{F}}_{\mathrm{stim}}}$, where ${\mathrm{N}}_{\mathrm{ch}}$ is the number of active channels, ${\mathrm{T}}_{\mathrm{pulse}}$ and ${\mathrm{T}}_{\mathrm{TBP}}$ are pulse width and time between pulses in ms, respectively, and ${\mathrm{F}}_{\mathrm{stim}}$ is pulse frequency in Hz. For example, for ${\mathrm{T}}_{\mathrm{pulse}}$ = 0.5 ms, ${\mathrm{T}}_{\mathrm{TBP}}$ = 1 ms, and ${\mathrm{F}}_{\mathrm{stim}}$ = 100 Hz, maximally five channels can be simultaneously active.

#### EMG recording

The EMG unit is based on an integrated circuit (IC) ADS1299 (Texas Instruments, Texas, USA), which consists of eight differential 24-bit sigma delta analogue-to-digital converters with integrated programmable gain amplifiers and a flexible input multiplexer. ADS1299 is fully programmable through the SPI communication interface. In the MaxSens design, it can be configured to use up to eight differential or single ended referenced inputs. An integrated driven-right leg amplifier is used as the active neutral electrode. The EMG unit is galvanically isolated from the electrical stimulation unit which allows it to be used safely during electrical stimulation. The gains can be set to 1, 2, 4, 8, 12 or 24 and a sampling frequency to 250, 500, 1000 or 2000 Hz.

#### Power supply

The input power source is a single cell Li-Poly battery with the capacity of 560 mAh. The power supply unit generates + 3.3 V for the microcontroller, + 50 V for supplying the current controlled H-bridge, galvanically isolated power supply for the EMG unit and battery charging through the USB port.

#### Stimulation and recording electrodes

**Fig. 3:**
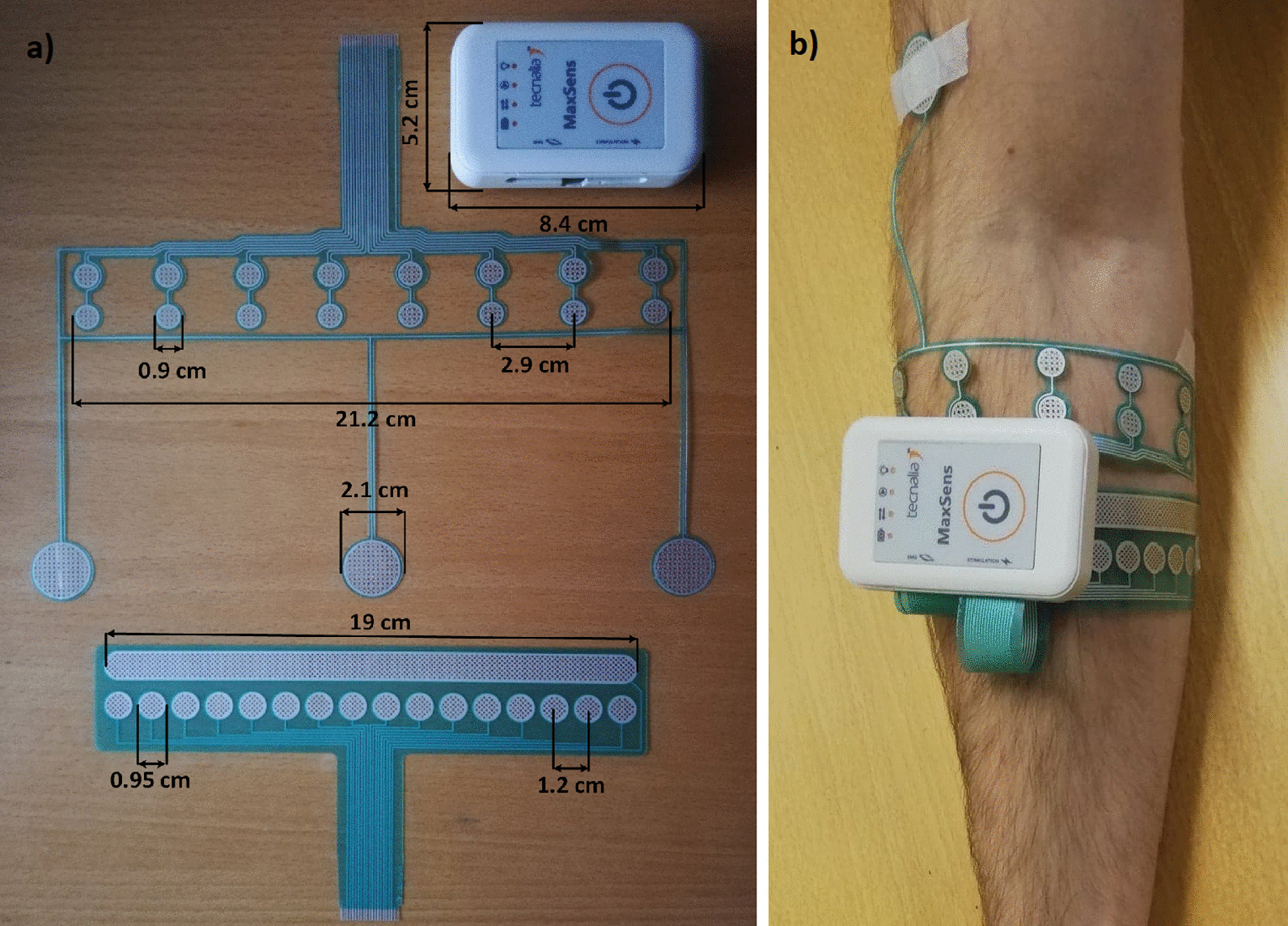
The image **a** shows the dimensions of the MaxSens system and of the recording (top) and stimulation (bottom) electrode, where **b** shows the placement of EMG electrode (proximal) and stimulation electrode (distal). In the actual application, an elastic band was applied over the electrodes to secure the electrodes and improve contact with the skin, while the MaxSens system was placed on the top of the band secured by a Velcro attached to the bottom of the stimulator

The custom-designed EMG and stimulation electrodes are shown in Fig. 3a. The EMG electrode comprises eight pairs of circular pads for bipolar EMG recording and three circular reference pads. Both electrodes were produced by screen-printing biomedical conductive Ag/AgCl and dielectric inks over 125 µm thick PET film. All electrode pads were covered with conductive hydrogel (AG725, Axelgaard, Denmark) to improve the electrode–skin contact.

### System operation

#### Firmware

The firmware for the embedded system was written in C programming language using Microchip Technology’s Atmel Studio 7 integrated development platform. A communication, stimulation and EMG recording task are executed independently and communicate with each other using the microcontroller’s hardware resources and interrupt driven firmware organization.

The communication task is responsible for the bidirectional Bluetooth and USB communication and receives textual commands from a host PC specifying stimulation and recording parameters. The received commands are then interpreted and responded to through bi-directional data exchange between the communication task and the two other tasks (namely, stimulation and EMG recording). When the commands for setting up the stimulation parameters are received, the parameters are checked, and only if they are internally consistent with the other parameters (see Section Electrical stimulation), the command is acknowledged (responding with “OK”), otherwise, it is rejected (responding with “ERR”).

The stimulation task is responsible for stimulus generation. Based on the stimulation parameters received from the communication task, the stimulation task controls the stimulus generation units in precisely defined time intervals, and sends the stimulation parameters to the EMG recording task to control the blanking algorithm, which is explained in the next section.

The EMG recording task adjusts the amplifier parameters, sampling frequency, gain for each channel and input electrode configuration (differential or single-ended reference) according to the parameters received from the communication task. The EMG amplifier blanking period and the onset of stimulation are received from the stimulation task in order to execute the stimulation blanking algorithm. Finally, this task transfers the collected EMG samples to the communication task so that they can be transmitted to the host PC. Importantly, the control of stimulation and recording consumes negligible MCU time (max 4% in the “worst” case) as these functions are performed in the background by dedicated peripheral modules. For instance, to generate an edge of a stimulation pulse, the MCU needs only to configure the compare module in response to an interrupt. Similarly, once the EMG signals are sampled by the EMG unit (ADS1299 IC), the MCU receives an interrupt to collect the data via the SPI interface. These interrupt routines are of highest priority, they are almost instantaneous, and hence most of the MCU time is in fact available for other tasks (e.g., parsing protocol messages).

#### Dynamic blanking

In order to suppress stimulation artifacts in EMG recordings, a novel blanking technique was implemented. Unlike commonly used methods based on hardware solutions in which the EMG amplifier inputs are shorted, the proposed technique is based on a firmware solution. The EMG amplifier is continuously active, even during stimulation pulse generation, and the number of EMG samples which need to be blanked are simply replaced with the last sample acquired before the blanking period (sample-and-hold technique). This approach is possible because of the EMG amplifier characteristics: DC amplifier topology, relatively low gain (up to 24) and a very short recovery time after amplifier saturation. Additionally, the implemented overvoltage protection circuits provide adequate amplifier inputs protection against overvoltage caused by the stimulation.

This blanking technique requires precise synchronization between the EMG unit and the electrical stimulation unit by the MCU. The blanking starts at the same time as the leading edge of the stimulation pulse and the period in which the EMG amplifier has to be blanked is computed before pulse generation based on the electric charge delivered by the pulse (amplitude × duration). The period is expressed as the number of blanked samples (NBS) of the EMG signal. The relation between NBS and the parameters of the stimulation pulse was experimentally derived. The tests were conducted using two adjacently placed EMG and stimulation channels since this positioning leads to strongest artefacts. The stimulation frequency was set to 50 Hz, the EMG amplifier gain to 24 (maximal value) and the sampling frequency to 2000 Hz. The pulse width was increased from 200 to 1000 µs in steps of 50 µs and, for each pulse width, an amplitude sweep from 1 to 10 mA in steps of 1 mA was performed. For each combination of pulse width and amplitude $({T}_{pulse}, {I}_{pulse})$, the duration of the generated stimulation artifact was measured as the number of corrupted EMG samples. The experimental data relating the pulse width and amplitude to NBS were fitted using a first order linear polynomial surface model: $\mathrm{z}\left(\mathrm{x},\mathrm{y}\right)= {\mathrm{p}}_{00}+{\mathrm{p}}_{10}\mathrm{x}+{\mathrm{p}}_{01}\mathrm{y}+{\mathrm{p}}_{11}\mathrm{xy}$, where (x,y) are pairs T_pulse_ and I_pulse_, and z(x,y) is NBS (Matlab function “fit” with the model type “poly11”). The coefficients were estimated from 95% confidence intervals. The polynomial model was then expressed in the product from, shown in Eq. (1), as that form was more convenient for embedded implementation. The resulting expression is:

1 $$NBS = \left( {0.25 \cdot I_{{pulse}} + 7.5{\text{ mA}}} \right) \cdot \left( {0.6 \cdot T_{{pulse}} + 500\,\upmu {\text{s}}} \right) \cdot \frac{{F_{{EMG}} }}{{10^{6} \,{\text{mA}}}}$$

where ${I}_{pulse}$ represents the amplitude of the stimulation pulse in mA, ${T}_{pulse}$ is the stimulation pulse duration in µs, ${F}_{EMG}$ is the sampling frequency of the EMG amplifier, and $NBS$ represents the number of EMG samples to be blanked (i.e., replaced by the value of the last sample recorded before stimulus generation). Therefore, increasing the current amplitude and pulse width will prolong the blanking period. Higher stimulation frequency will increase the number of blanked samples. The Eq. (1) was tested on all the recording/stimulation electrode pairs, and the validity of the equation was thereby confirmed.

However, the Eq. (1) is determined using a single active channel. When multiple stimulation channels are active, multiple pulses will be delivered within a single stimulation period separated by inter-pulse intervals (asynchronous protocol, as described above). In this case, the blanking will start with the first pulse and include the biphasic pulse width and inter-pulse interval of all pulses apart from the last pulse in the sequence (blue area in Fig. 2). The blanking period related to the last pulse will be computed according to Eq. (1) (green area in Fig. 2). The NBS in case of multiple active channels is therefore given by:

2 $$NBS = \left[ {\left( {0.25 \cdot I_{{pulse}}^{N} + 7.5{\text{ mA}}} \right) \cdot \left( {0.6 \cdot T_{{pulse}}^{N} + 500\,\upmu {\text{s}}} \right) + \sum\limits_{{i = 1}}^{{N - 1}} {\left( {2 \cdot T_{{pulse}}^{i} + T_{{TBP}}^{i} } \right)} } \right] \cdot \frac{{F_{{EMG}} }}{{10^{6} \,{\text{mA}}}}$$

where ${I}_{pulse}^{N}$ and ${T}_{pulse}^{N}$ represent the amplitude and duration of the last stimulation pulse in mA and µs, respectively, ${T}_{pulse}^{i}$ represents the duration of a single phase of the *i*th pulse in µs, while ${T}_{TBP}^{i}$ represents the inter-pulse interval time between the *i*th and (*i* + *1*)th pulse in µs. The parameter ${F}_{EMG}$ represents the sampling frequency of the EMG amplifier, and $NBS$ is the number of blanked EMG samples. Thus, increasing the number of active stimulation channels and/or inter-pulse interval will increase the NBS.

## Experimental assessment

Ten subjects (eight males and two females, right-handed with a mean age of 27.1 ± 2.4 years) were recruited to test the performance of simultaneous recording and stimulation. All subjects participated voluntarily and had no musculoskeletal or skin disorders. Before participating in experiments, the subjects read and signed a consent form and were informed about the study’s methods and objectives. The study was conducted in accordance to the Declaration of Helsinki and approved by the Ethical committee of the Nordjylland Region, Denmark (approval number: N 20 150 075).

### Experimental setup

Figure 3b shows the electrode placement and their connection to the MaxSens system. The electrodes are flexible and thus the end of the electrode is bent and plugged into flat ZIF connectors. The stimulator and the electrodes were placed on the dominant forearm. The EMG electrode was positioned circumferentially around the forearm approximately 3 cm distal to the elbow crease. The reference pads were placed on the condyle of the elbow joint and one distally on the upper arm. The forearm was not shaved nor prepared in any other manner for the EMG recording to best mimic the envisioned application. The stimulation and recording electrodes were placed circumferentially to make the setup more compact. Similar array configurations have been used before [27, 44, 45]. Importantly, MaxSens system is not specific to the electrode type used in the present study and it could be connected to electrodes of different shapes, sizes and pad configurations (e.g., matrix versus array). Sports tape and elastic sports bands were wrapped around the electrodes to ensure good electrode–skin contact and to have a stable surface on which to attach the Velcro-underside of the stimulator.

During the experiment, the subject was seated in a comfortable chair with the dominant forearm held vertically down along the side of the body. A 14″ monitor laptop PC was placed on a table approximately 50 cm from the subject, and was used to provide visual feedback in the experiment. The PC controlled the stimulation and received the recorded EMG signals via a USB connection to the MaxSens system. The online control loop and experimental tasks were programmed in Matlab 2019b (MathWorks, Massachusetts, USA).

### Signal processing and myoelectric control

The signal processing pipeline was implemented on the host PC to control a cursor (virtual prosthesis) along two degrees of freedom, as in [28]. The MaxSens system acquired the EMG data and streamed them to the host PC, which translated myoelectric control signals into  cursor movement and sent stimulation commands to MaxSens system providing tactile feedback to the subject.

**Fig. 4:**
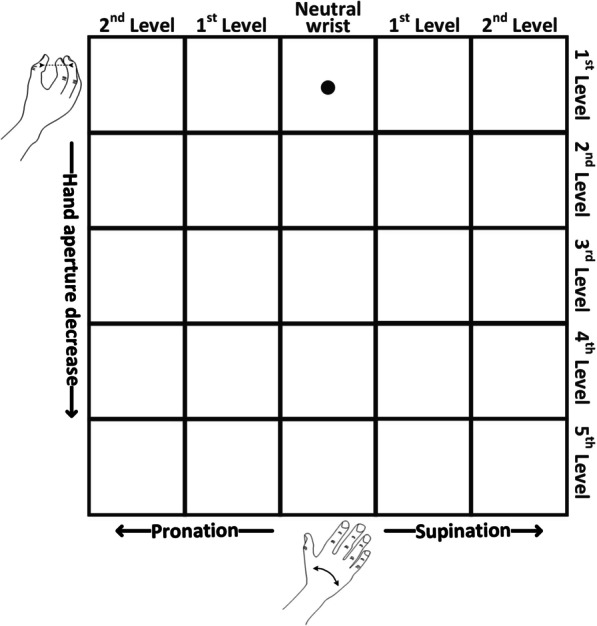
Illustration of the grid used in the planar target reaching task where the horizontal and vertical axes corresponded to wrist rotation and hand aperture, respectively. The black cursor is located at the initial position, which indicates a neutral hand configuration, i.e. neutral wrist and hand fully opened. The cursor moved continuously in response to subject’s myoelectric commands while the grid cells indicated the ranges in each DoF that were mapped to the levels of discrete feedback (see text for details)

The hand opening and closing, wrist pronation and supination and rest, as well as contraction intensity, were estimated from the EMG and mapped to up, down, left and right movement of the cursor, while the movement velocity was proportional to the contraction intensity. This control scheme was selected because it can be used to control a hand prosthesis with an active wrist (hence two DoFs). Figure 4 shows a planar area within which the cursor was moved and the mapping between the movement classes and cursor directions. To further approximate the practical use of a prosthesis, the maximum velocity of the cursor was set to the maximum rotation velocity of Michelangelo Hand (Otto Bock, Duderstadt, Germany), i.e., the full rotation is performed in approx. 3 s.

The EMG signals were acquired with a sample rate of 2 kHz and transmitted to the host PC using a USB connection. Channel 5 was excluded from the data acquisition as it recorded no signal. The EMG signals were band-pass filtered between 15 and 350 Hz using a 2nd order Butterworth filter. Notch filters were applied to remove power line noise at 50 Hz and its two harmonics. Finally, a 10-sample Hampel filter was applied to remove outlier values, which were present occasionally during stimulation. The commonly used time domain features, namely, mean absolute value, zero crossings, slope sign changes and waveform length, were extracted [46]. The features were extracted in windows of 300 ms with 100 ms overlap, in order to yield a high classification accuracy with a fast update time [47]. The linear discriminant analysis (LDA) was used for classification while multiple linear regression models (one per class) were applied to estimate muscle activation level, providing thereby sequential and proportional control along the two DoFs [34, 48–51].

During online control with the stimulation active, the EMG signals contained blanked segments. All the blanked segments were identified and removed, as recommended in [37], and hence only the “useful” data were considered in each window, thereby decreasing the effective window length. As explained in [37], this approach maintains classification accuracy while preserving the update time.

### Electrotactile stimulation

To address the specific aim of the present study, the electrotactile feedback was designed to maximally stress the myoelectric pipeline, while still transmitting to the subject purposeful and intuitive information that is useful for closed-loop control. The feedback scheme therefore included both amplitude and frequency modulation since increasing these two parameters produces stronger and more frequent stimulation artefacts and therefore longer blanking (cf. Eq. (1)). In addition, both parameters were modulated through the full range that is relevant for practical applications. As described below, the amplitude was changed from sensation to discomfort threshold to produce clearly perceivable sensations that were not uncomfortable. The maximum frequency was set to 50 Hz since this value evokes a fused sensation, and it becomes more difficult to discriminate the frequencies that are higher. Finally, spatial modulation, in which the information is coded by activating different electrodes of the array, is also included in the scheme so that the stimulation is delivered in the vicinity of all the recording electrodes.

**Fig. 5:**
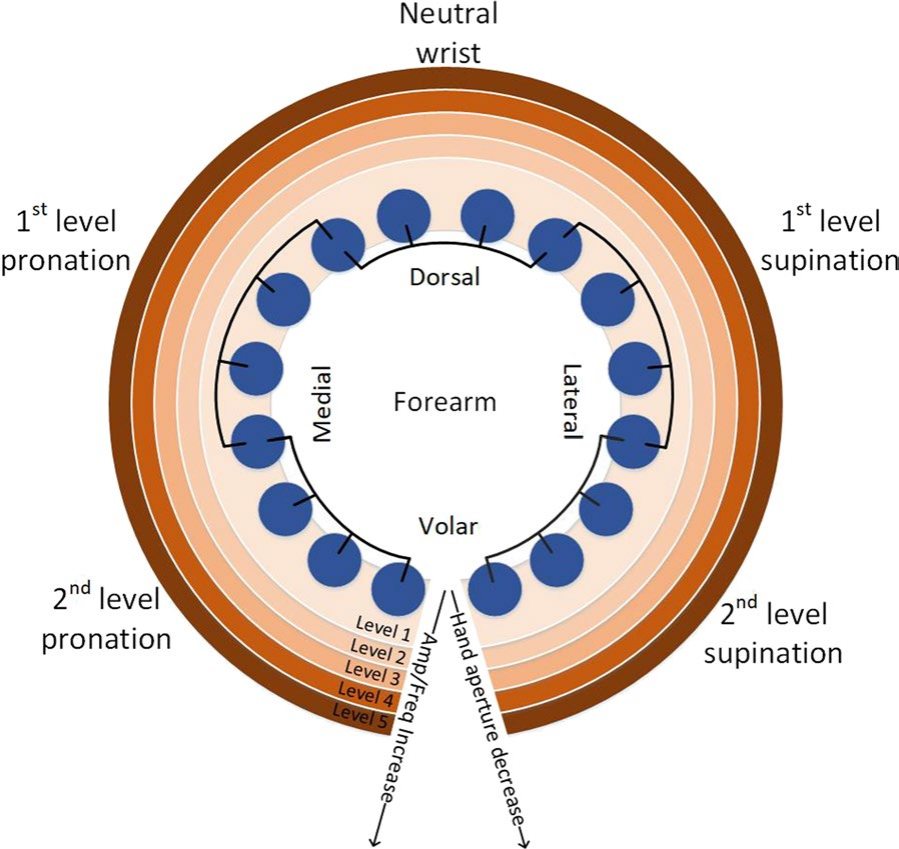
Illustration of the developed feedback-coding scheme. The blue circles represent electrode pads, which are connected in groups activated together (black lines), and the orange toned rings indicate the levels of amplitude and frequency. The level of wrist rotation was communicated by activation of four neighboring pads. Supination and pronation were conveyed by activating groups of pads on the lateral and medial side of the forearm, respectively. A decrease in hand aperture was communicated by a simultaneous increase of the amplitude and frequency of the currently active group of pads. No stimulation was provided at neutral position, i.e. neutral wrist and hand fully opened

The designed feedback scheme provided information regarding hand aperture and wrist rotation (see Fig. 5). While the control was continuous, as explained in the previous section, the feedback transmitted discrete information; the approach adopted in several other studies [27, 28, 52–54]. Each DoF was divided into five intervals. In Fig. 4, this is indicated visually by dividing the area into a grid, where each cell corresponds to a combination of intervals in the two DoFs. One cell indicated the neutral position (neutral wrist and hand fully opened), eight cells corresponded to the movement along a single DoF (four for hand aperture and four for wrist rotation), while sixteen remaining cells indicated a combination of the two DoFs (both DoFs outside the neutral position). No stimulation was provided when the cursor was located in the cell indicating neutral position. Information on the wrist rotation was provided by sequentially activating the groups of 4 pads to mimic the direction of the rotation movement. Therefore, the subject perceived a sensation over a relatively large area (4 pads simultaneously active) that would rotate medially during wrist pronation and laterally during wrist supination. The assumption was that the spatially congruent mapping would facilitate the interpretation of feedback.

The decrease in the hand aperture was conveyed as an increase in current amplitude and stimulation frequency in the four active pads, thus, mimicking a more tightly closed hand. The frequency was modulated from 10 Hz in the first interval (indicating hand fully open) to 50 Hz in the fifth interval (closed hand) using steps of 10 Hz. The stimulation amplitude was changed between sensation threshold at the first interval to just below the discomfort threshold at the fifth interval. The amplitude values for the intermediate intervals were equidistantly separated in the range between the first and fifth interval. In this way, the amplitude values were maximally separated within the dynamic range to facilitate discrimination of the feedback levels by the subject. Although MaxSens system allows fine modulation of the pulse amplitude and frequency, 5 discrete levels were used in the present study to allow the subject to interpret the feedback easily, i.e., he/she needs to memorize “only” five distinct sensations, which can be achieved after a brief training [55]. The pulse width was constant and set to 300 μs. The pulse width was selected based on our previous experience to allow fine modulation in the intensity of elicited sensations while still ensuring that the discomfort threshold can be reached in all subjects when changing the pulse amplitude. Therefore, the subject perceived the sensation that would become stronger and faster as the virtual hand was closing. The assumption was that simultaneous change in both parameters (double cuing) would assist the subjects in discriminating the levels and interpreting the feedback.

### Experimental protocol

The experimental protocol for the assessment of closed-loop myoelectric control was similar to that described in [28] and it will be summarized hereafter. The system was placed on the subjects as explained in Section Experimental setup. The psychometric parameters (sensation and discomfort thresholds) were determined for each channel. The subjects then performed a maximum long-term voluntary contraction (MLVC), where they have been asked to contract their muscle strongly but at the level that can be maintained for 15 s without getting fatigued [56]. To collect the EMG data for the training of the myoelectric classifier, the subject was asked to perform each movement while tracking a trapezoidal trajectory with a cursor moving horizontally with time and vertically relative to the subject’s contraction intensity [57]. The contraction level was estimated by averaging the mean absolute value of the windowed EMG signal over all channels. The trajectory consisted of a 3-s incline, a 5-s plateau and a 3-s decline. Three recordings of trajectory tracking were acquired per movement class, with plateaus at 40%, 50% and 70% of the MLVC. Lastly, a 15-s recording of the rest class was acquired. Pilot tests showed a slightly higher EMG baseline when the stimulation was activated compared to no stimulation. Therefore, the data for the rest class was recorded while eight stimulation pads were activated (two active pads interleaved by two inactive pads etc.) at just above the sensation threshold.

After training the classifier, the subjects performed the experimental task. First, the subjects practiced the myoelectric control, after which they learned to understand the feedback scheme. Lastly, the subjects practiced the myoelectric control combined with tactile feedback (closed-loop control). To teach the subjects how to interpret the feedback coding, they went through familiarization and reinforced learning. In the familiarization phase, the visual and tactile feedback indicating movement along single DoFs was presented to the subject. Hence, he/she could associate the visual cue to the tactile sensation. In the reinforced learning phase, the subject was blinded and presented with the feedback for each combination of DoF intervals (all grid cells), and then asked to guess the indicated intervals. The subject was informed about the correct answer following a wrong estimate. The reinforced learning was finished after all grid cells were presented twice (two times 24 trials). The feedback training lasted approximately 15 min per subject.

After this brief training, the subjects performed target-reaching tests in three conditions [45, 58–61]. The first condition was the benchmark assessment, where only visual feedback was provided. The grid shown in Fig. 4 was displayed on the screen and the target cell was indicated in red. As explained before, the cursor moved continuously while the visual feedback indicated the cell in which the cursor was located (discrete feedback). If the subject reached the target cell within 30 s and remained in the target for 2 s (dwell time), the task was accomplished successfully, otherwise it was counted as a failure. In the second condition (“combined feedback”), the electrotactile feedback was delivered together with visual feedback to evaluate whether the stimulation affected the control performance. In this condition, the assumption was that the subjects would primarily rely on vision, since tactile and visual feedback provided the same information, which was easier to interpret by looking at the screen compared to decoding the tactile patterns. Hence, the electrical stimulation in this case served mainly as an interference to the EMG recording. In the last condition, only electrotactile feedback was provided, to assess whether the feedback was clear and intuitive enough for the subject to interpret it and use it for control. This assessment was performed twice using a different sequence of target cells in each run. The benchmark test was conducted after training the myoelectric control. The subject performed the other two tests after practicing the feedback scheme. In each test, all grid cells were used as targets except for the cell corresponding to the neutral position (neutral wrist and hand fully opened), hence 24 targets per test in total. The total experiment duration was between 2 and 3 h.

### Data analysis

The outcome measures were completion rate, time to reach a target, path efficiency and the number of overshoots. These are common measures to evaluate the quality of control in target-reaching tasks [45, 58], and they were also used in our previous study [28]. The completion rate was the percentage of successful trials. The time to reach a target was the time in seconds from when the trial started until the target was reached including the dwell time. The path efficiency was computed by dividing the lengths of the optimal and generated path. For single-DoF targets, the optimal path was a straight line from the starting point to the border of the target, and for combined-DoF targets, it was the shortest path to the nearest corner of the target from the starting point using horizontal and vertical lines only. The number of overshoots was counted as the number of times the cursor entered a target but then moved out before the dwell time expired. This measure was used to assess the stability of control (e.g., potential drift in control due to interfering electrical stimulation) and it is reported as the mean number of overshoots per trial.

The outcome measures were calculated for each trial, after which the results were averaged within subjects for each evaluation test. As there was no significant difference in performance between the two tests with tactile feedback only, the respective within-subject outcome measures were computed as the average of the results achieved in the two tests. Only successful trials were considered relevant for the analysis, and therefore, unsuccessful trials were left out in the computation of all outcome measures besides the completion rate. Non-parametric statistics were employed as the data showed not to be normally distributed following a Kolmogorov–Smirnov test. A Friedman test was applied to assess significant differences between the test conditions, and a Tukey–Kramer test was used for post-hoc pairwise comparison. A significance level was set to *p* < 0.05. The results in the text are reported in the form of median/interquartile range (IQR).

## Results

### Effect of dynamic blanking algorithm

**Fig. 6:**
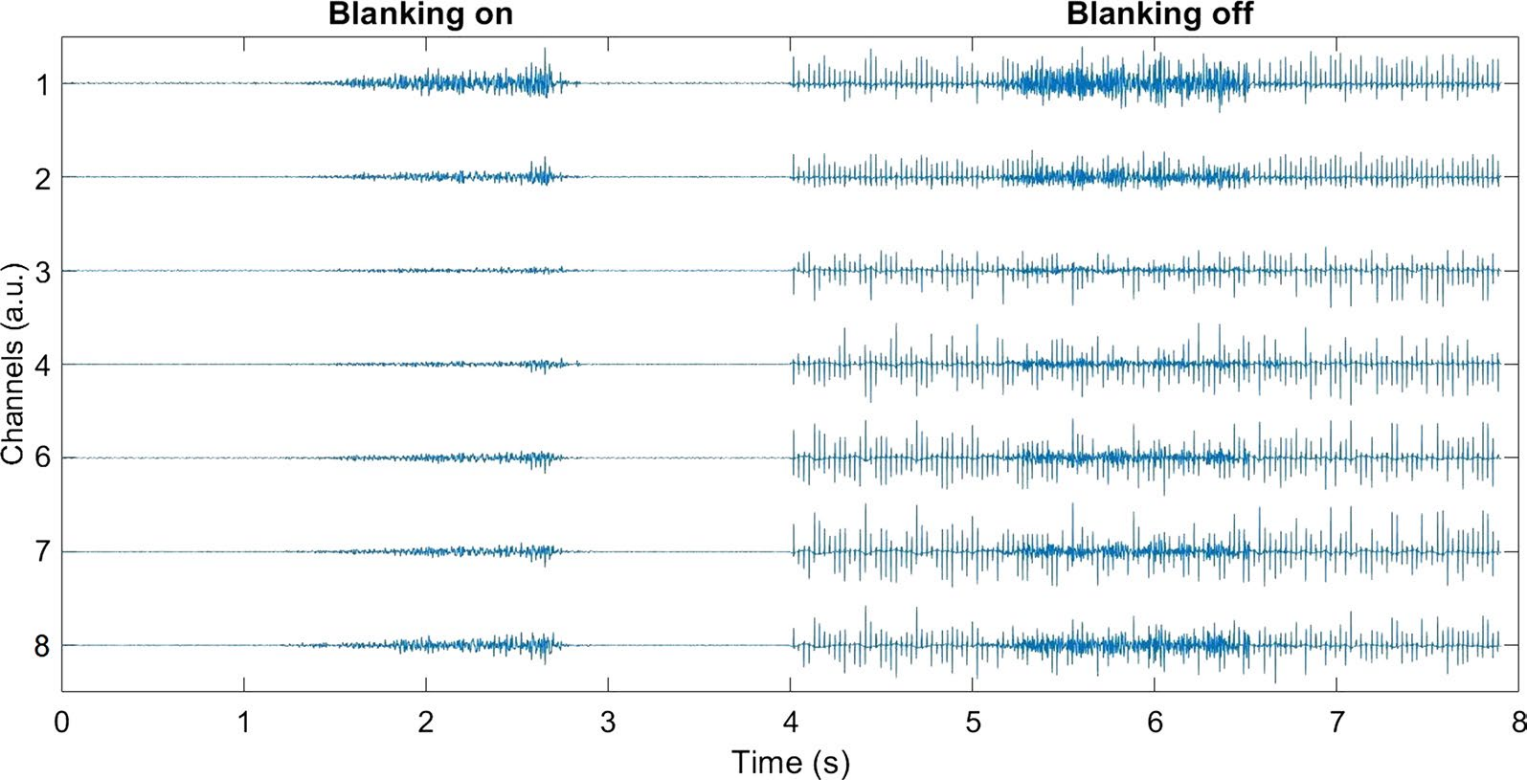
EMG recorded when the subject performed hand closing with concurrent electrotactile stimulation. The signals are reported in arbitrary units. In the left recording, the blanking was activated, while it was inactive in the right recording. The recording without blanking was corrupted with pronunced artifacts that are larger in magnitude than the generated EMG

Figure 6 shows an example of the data collected using the developed system while the subject performed hand closing. Before plotting, the signals were filtered as explained in Section Signal processing and myoelectric control, and they are reported in arbitrary units. While the subject performed the movement, the electrotactile stimulation was delivered at amplitude level 3, pulse width of 300 µs and frequency of 30 Hz with 8 channels active as in the baseline EMG acquisition (see Section Experimental protocol). The figure indicates that the system can record EMG of good quality, with contraction bursts that were clearly visible and different across channels. However, the electrotactile stimulation substantially corrupted the recorded EMG signal. The induced artifacts were large and with variable amplitude, but they disappeared when the blanking was activated. Therefore, the dynamic artefact-blanking successfully eliminated the stimulation interference. Nevertheless, the stimulation did introduce some low-amplitude artifacts in the recording, which resulted in a higher amplitude of the baseline EMG. Specifically, during hand resting without stimulation, the baseline EMG was approximately four times lower than with the stimulation (6.4% ± 5% normalized to MLVC amplitude of each subject vs. 1.7% ± 0.9% without stimulation).

**Fig. 7:**
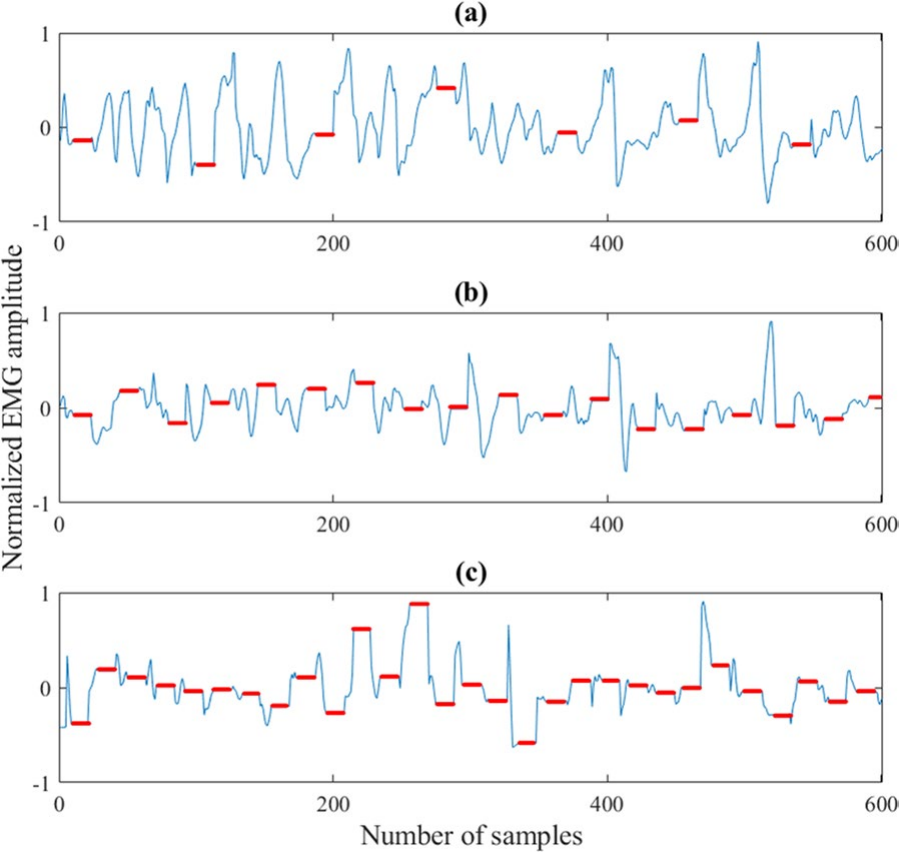
300 ms windows of EMG recorded while the stimulation was delivered at three different frequencies: **a** shows a recording made using 20 Hz frequency, level 2 amplitude, 300 µs pulse width and four active channels, while in **b** the frequency was changed to 50 Hz and in **c** to 80 Hz. The red lines indicate blanked segments of the signal. In these segments, the EMG value was constant since it was “frozen” by the sample-and-hold (see Section Dynamic blanking)

Figure 7 illustrates how frequency of stimulation affects the occurrence of blanking periods within a single window of EMG data (300 ms/600 samples). In the first recording, (Fig. 7a), parameters were set to level 2 amplitude (~ 2 mA), 20 Hz frequency, 300 µs pulse width and four channels were activated with a 100 µs inter-pulse interval. This resulted in approximately 14 blanked samples per stimulation pulse, which agrees with Eq. 2. In a single window, 7 segments of blanked data (constant value due to sample-and-hold) occurred leading to a loss of 16% of EMG data. In the second and third recordings (Fig. 7b and c), the frequency was increased to 50 Hz and 80 Hz, respectively, which resulted in 18 and 29 blanked segments within a single window corresponding to a loss of 39% and 64% of EMG data, respectively. Calculating the number of blanked samples for the feedback coding scheme used in the present study (Fig. 5), the data loss was in the range of ~ 7 to ~ 40% per window when moving from hand fully open to hand fully closed.

### Experimental task

**Fig. 8:**
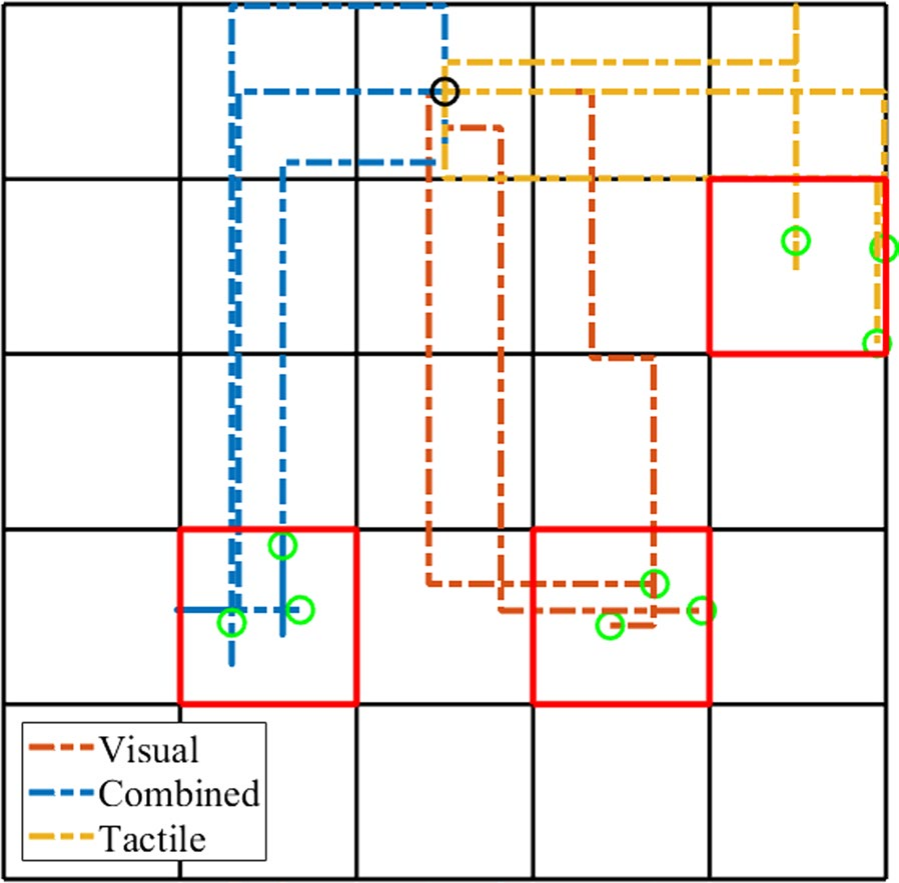
Examples of trajectories generated while using visual (brown traces), combined (blue traces) and tactile feedback (yellow traces). The black circle indicates the starting point, the green circles indicate end points when the target was reached and the red squares are the targets

Example trajectories from the three feedback conditions can be seen in Fig. 8. The traces were smooth for all feedback conditions with only few path corrections indicating that the myoelectric control was not affected by the electrical stimulation.

**Fig. 9:**
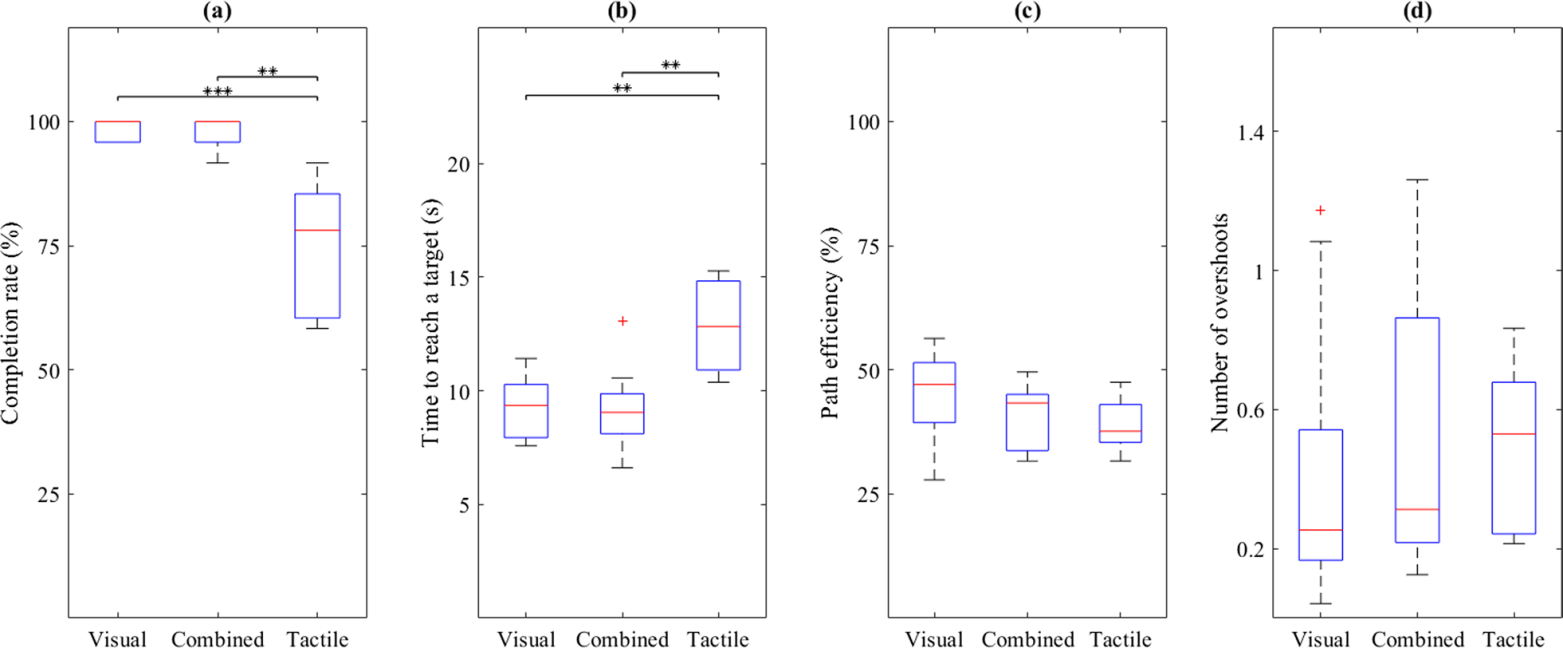
Box plots of the outcome measures obtained in the visual, combined and tactile feedback conditions: **a** completion rate, **b** time to reach a target, **c** path efficiency and **d** the number of overshoots. The red lines, blue boxes and black whiskers represent medians, interquartile ranges and maximum and minimum values, respectively, while the red crosses are outliers. The asterisks indicate significant differences (* is *p* < 0.05, ** is *p* < 0.01 and *** is *p* < 0.001)

A summary of the results characterizing the quality of closed-loop myoelectric control in the three feedback conditions are shown in Fig. 9. There was no significant difference between the visual and combined feedback condition in any outcome measure and in both conditions the subjects achieved high completion rates (median/IQR of 100/4% for visual and 100/4% for combined). Seven subjects successfully reached all the targets in the visual and combined conditions, respectively.

As expected, the performance in the tactile feedback condition was worse compared to that achieved using visual and combined feedback. The decrease in performance was statistically significant in completion rate (78/25%) and time to reach a target (13/4 s). The median path efficiency (38/8%) was lower and the number of overshoots (0.5/0.4 overshoots) higher, but the difference in these two cases was not statistically significant. The completion rate of the target-reaching task with tactile feedback and the success rate in recognizing the tactile codes in the reinforced learning (74/13%) were not significantly different (Wilcoxon signed rank test).

## Discussion

In this study, we presented and evaluated a novel compact electronic interface with an embedded dynamic artefact blanking algorithm to allow simultaneous EMG recording and electrotactile stimulation on the ipsilateral forearm. To the best of our knowledge, this is the first embedded system in the context of online closed-loop control of hand prostheses using electrotactile stimulation, where multichannel recording and stimulation electrodes can be placed in close proximity without signal distortion and therefore potentially integrated in a prosthetic socket. The closed-loop myoelectric control and the effectiveness of the blanking algorithm was evaluated in a target-reaching task, in which both visual and electrotactile feedback were provided, and the performance was compared to that achieved in the benchmark condition with only visual feedback. Furthermore, the degree to which the tactile feedback could be exploited for control was evaluated in a condition where only electrotactile stimulation was delivered to the subject.

Importantly, the performance in visual and combined feedback conditions was similar in all outcome measures. A high completion rate was expected with the visual feedback, and the fact that it did not change with the addition of electrotactile stimulation demonstrates that myoelectric control was not affected by the stimulation. This does not automatically mean that the control was not disturbed, as the subjects could have used visual feedback to compensate for the disturbance and maintain similar success rate. However, the fact that the time to reach a target and path efficiency did not change significantly implies that the blanking algorithm was indeed efficient and that the loss of information due to blanking did not disturb the classification performance (Figs. 8 and 9).

Nevertheless, the stimulation did produce an increased baseline EMG amplitude. However, the baseline shift was still low, and did not affect the control performance as the rest class could be activated reliably by the subjects. The change in the baseline was easily accommodated by performing an additional recording with the stimulation active, as described in Section Experimental protocol. Concentric electrode configurations reduce the current spread and could, therefore, be a method to suppress this change in the baseline [25].

Another constrain introduced by the blanking algorithm was the loss of information (blanked samples). As demonstrated in Section Effect of dynamic blanking algorithm, the loss increases substantially with frequency, and effectively no data would remain (all samples blanked) for the frequencies > 132 Hz, when using the same parameter settings as for the recording in Fig. 7. Therefore, despite the stimulator can generate the pulses at up to 400 Hz, the effective maximum frequency is far lower when the stimulation is combined with simultaneous recording of EMG. This is however not a critical limitation. The present results demonstrated that the frequency can be safely increased to 50 Hz without affecting the performance, and this is still within a preferred range of frequencies for electrotactile stimulation [62].

The conditions with visual and combined feedback are not enough to prove the applicability of the system, as the electrotactile stimulation could have been too weak to produce a significant interference. Therefore, the condition with the tactile feedback only was included in the protocol to demonstrate that the feedback was meaningful for the subjects. And indeed, the target-reaching test with only tactile feedback showed a good completion rate (~ 80%) and no significant difference compared to the visual and combined conditions for the path efficiency and the number of overshoots. These results confirm that the feedback could be successfully exploited by the subjects to efficiently navigate and maintain the target DoF configuration. Significantly lower completion rate and longer time to achieve a target with respect to visual feedback was an expected result. Similarly, it was not expected that adding stimulation (tactile feedback) would improve performance when visual feedback was also available. Indeed, the dominance of visual over tactile information is a well-known challenge in the design of effective somatosensory feedback interfaces in upper limb prostheses [8, 63]. However, the aim of the present study was to validate the system by demonstrating that it can record EMG while eliciting clear and meaningful tactile sensations. Investigating the potential benefits of such feedback will be addressed in future work. In general, the tactile feedback is likely to be beneficial when the vision is impaired or unavailable, as in the tactile only condition of the present study, and/or when the tactile channel conveys variables that are not directly observable by vision (e.g., EMG biofeedback [64]). Importantly, the present study showed that MaxSens device is effective and versatile enough to implement such closed-loop scenarios. For instance, in the present experiment, the system conveyed proprioceptive information, but other variables could be similarly mapped to the electrode array (e.g., contact [65], grasping force [11], myoelectric signal [54]), leading to a clinically useful feedback.

A specific advantage of electrotactile stimulation is the ability to change the parameters independently leading to a rich repertoire of coding schemes to transmit feedback information. The feedback encoding used in the present study modulated the current amplitude, stimulation frequency and spatial activation of electrode pads to communicate two feedback variables (aperture and rotation). As explained before, the aim was to stress the myoelectric system with different types of disturbances and the results demonstrated that the myoelectric control was robust. This implies that the presented system can be used to implement all relevant encoding approaches (i.e., parameter and spatial modulation) in different combinations or individually, without affecting the EMG recording and myoelectric control. This is an important conclusion for the applicability of the developed system to different closed-loop myoelectric scenarios.

For instance, the system could be used to implement two feedback mappings presented in [28], which were based on amplitude and spatial modulation, respectively, while the frequency was constant. These feedback schemes were evaluated using the same test as in the present study, resulting in the median completion rates that were significantly better than in the current experiment (94/10% and 94/2% versus 78/25%). However, those coding schemes where exclusively designed to be intuitive, while the feedback in the present study was constructed to challenge the myoelectric control. Contrary to [28], where the stimulation and recording were performed by two different systems placed on separate arms, the present assessment used a single unit. Therefore, this shows that proprioceptive information can be communicated intuitively via electrotactile stimulation, while activating the muscles and recording myoelectric signals from the same arm.

Some subjects reported a change in sensation quality and localization when performing movements, in particular rotation, although the stimulation parameters were constant. This change in sensation would occasionally misguide the subjects during the evaluation test with no visual feedback. However, the overall impact on performance was likely small since there was no significant difference between reinforced learning (stimulation without muscle activation) and target reaching test. A possible reason for the change in sensation could be that different skin afferents were activated by the stimulation when rotating the wrist or that the electrode–skin adhesion was altered [66]. This could be also an additional contributing factor for higher performance in [28], where the sensation was not disturbed by muscle activation and forearm movements (due to the contralateral placement of recording and stimulation unit). Although this effect is likely to be smaller for amputees, it should be taken into consideration when future feedback schemes are designed for simultaneous EMG recording and electrotactile stimulation on the ipsilateral forearm.

The assessment of the system was conducted in able-bodied subjects which is appropriate considering the aim and scope of the study. The goal was to demonstrate the functioning of the system and in particular the robustness of the dynamic blanking algorithm. To achieve this, the closed-loop control was tested in same subjects across different conditions. This demonstrated that the system successfully protected the myoelectric control from the impact of stimulation. While the performance in amputees might change, for instance, due to different sensitivity and/or control capability (e.g., the pattern-classification control might be more sensitive to information loss), the performance ratio between the conditions and thereby the overall conclusion would likely remain the same. The next step in this development is the functional evaluation with the prosthesis and in this case, the participation of target users will be indeed essential. The developed electronic interface is compact and both electrodes are slim and flexible, and could be therefore easily integrated in a prosthetic socket. The added weight and interaction between the socket/splint and the forearm might influence the quality of control as well as the subjective perception of the electrotactile feedback.

Importantly, the developed system is equipped with a Bluetooth module, which allows direct connection to the state of the art prosthetic hands supporting the same interface (Michelangelo from Otto Bock and i-Limb from Össur). In the present study, the EMG data were streamed from the device to a laptop PC, where they were processed, but the embedded module have enough processing power to implement classification as well as feedback mapping within the firmware, possibly leading to a self-contained system [67].

## Conclusion

This study presented a novel compact system for simultaneous recording and stimulation with a dynamic artefact-blanking algorithm and evaluated its applicability for closed-loop myoelectric control. Specially designed, flexible and slim EMG recording and electrotactile stimulation electrodes were placed adjacently to each other on the dominant forearm. The feedback communicated proprioceptive information regarding wrist rotation and hand aperture, and was designed to stress the myoelectric control while remaining intuitive to interpret. The results indicated that the electrotactile stimulation did not affect the control quality and that the developed feedback coding scheme resulted in good control. The presented system is therefore an integrated solution for an effective closed-loop control using electrotactile stimulation, which supports all relevant coding schemes and can be possibly embedded into a prosthetic socket.

## Data Availability

The experimental and/or analysed datasets can be made available from the leading author upon reasonable request.
